# Variants in *GLIS3* and *CRY2* Are Associated with Type 2 Diabetes and Impaired Fasting Glucose in Chinese Hans

**DOI:** 10.1371/journal.pone.0021464

**Published:** 2011-06-29

**Authors:** Chen Liu, Huaixing Li, Lu Qi, Ruth J. F. Loos, Qibin Qi, Ling Lu, Wei Gan, Xu Lin

**Affiliations:** 1 Key Laboratory of Nutrition and Metabolism, Institute for Nutritional Sciences, Shanghai Institutes for Biological Sciences, Chinese Academy of Sciences and Graduate School of the Chinese Academy of Sciences, Shanghai, China; 2 Department of Nutrition, Harvard School of Public Health, Boston, Massachusetts, United States of America; 3 MRC Epidemiology Unit, Institute of Metabolic Science, Addenbrooke's Hospital, Cambridge, Cambridgeshire, United Kingdom; South Texas Veterans Health Care System, United States of America

## Abstract

**Background:**

Recent genome-wide association studies have identified a number of common variants associated with fasting glucose homeostasis and type 2 diabetes in populations of European origin. This is a replication study to examine whether such associations are also observed in Chinese Hans.

**Methods:**

We genotyped nine variants in or near *MADD*, *ADRA2A*, *CRY2*, *GLIS3*, *PROX1*, *FADS1*, *C2CD4B, IGF1* and *IRS1* in a population-based cohort including 3,210 unrelated Chinese Hans from Beijing and Shanghai.

**Results:**

We confirmed the associations of *GLIS3*-rs7034200 with fasting glucose (beta = 0.07 mmol/l, *P* = 0.03), beta cell function (HOMA-B) (beta = −3.03%, *P* = 0.009), and type 2 diabetes (OR [95%CI]  = 1.27 [1.09–1.49], *P* = 0.003) after adjustment for age, sex, region and BMI. The association for type 2 diabetes remained significant after adjusting for other diabetes related risk factors including family history of diabetes, lipid profile, medication information, hypertension and life style factors, while further adjustment for HOMA-B abolished the association. The A-allele of *CRY2-*rs11605924 was moderately associated with increased risk of combined IFG/type 2 diabetes (OR [95%CI]  = 1.15[1.01–1.30], *P* = 0.04). SNPs in or near *MADD*, *ADRA2A*, *PROX1*, *FADS1*, *C2CD4B, IGF1*, and *IRS1* did not exhibit significant associations with type 2 diabetes or related glycemic traits (*P*≥0.10).

**Conclusions:**

In conclusion, our results indicate the associations of *GLIS3* locus with type 2 diabetes and impaired fasting glucose in Chinese Hans, partially mediated through impaired beta-cell function. In addition, we also found modest evidence for the association of *CRY2*-rs11605924 with combined IFG/type 2 diabetes.

## Introduction

Genome-wide association studies (GWAS) have achieved great success in identifying common genetic variants associated with type 2 diabetes. However, most of the diabetes-related genetic variants identified by GWAS appear to influence insulin secretion rather than insulin resistance [1]. Recently, a GWAS of type 2 diabetes identified a type 2 diabetes risk variant (rs2943641, near *IRS1*) that was associated with insulin resistance and hyperinsulinemia in populations of European ancestry [2]. Remarkably, previous candidate gene association studies also observed that *IRS1*-rs1801278 (G972R, not in linkage disequilibrium with rs2943641) was associated with whole body insulin resistance [3], [4], but generated inconsistent results and not associated with type 2 diabetes [5]. More recently, a large genome-wide meta-analysis of data from 21 GWAS in populations of European origin identified a common variant near IGF1 associated with hyperinsulinemia and insulin resistance (HOMA-IR), and nine novel loci (in or near *ADCY5*, *MADD*, *ADRA2A*, *CRY2*, *FADS1*, *GLIS3*, *PROX1*, *SLC2A2*, *C2CD4B*) influencing fasting glucose [6]. The associations of *ADCY5* and *PROX1* loci with type 2 diabetes also reached genome-wide significance [6]. Each of these loci had a small effect on fasting glucose (0.008 to 0.030 mmol/l per allele), and in combination with the previously identified *G6PC2*, *MTNR1B*, *GCK*, *GCKR* and *DGKB-TMEM195* loci they explained only 4% of the variation in fasting glucose [6]. A replication study in the Danish population-based Inter99 cohort (5,722 non-diabetic individuals) confirmed that the *DGKB-TMEM195*, *ADRA2A*, *GLIS3*, *C2CD4B* and *PROX1* loci were significantly associated with reduced glucose-stimulated beta cell function [7]. More recently, another study in a case-control sample of Chinese also replicated the associations of *DGKB*, *MADD*, *GLIS3*, *PROX1* and *IGF1* with type 2 diabetes and/or glycemic traits [8].

However, most of the previous studies were conducted in case-control samples, and study in a general population has more potential to provide insight into mechanisms by which genetic variants contribute to type 2 diabetes. We therefore examined whether these novel loci identified by recent GWAS studies are associated with type 2 diabetes and diabetes-related traits in a population-based cohort of 3,210 unrelated Han Chinese from Beijing and Shanghai.

## Methods

### Study design

The study population for the present study consisted of 3,210 unrelated Chinese Hans (1,423 men and 1,787 women) aged 50–70 years from the Study on Nutrition and Health of Aging Population in China. The study design and protocol has been described in detail previously [9]. Briefly, the study was implemented simultaneously in both geographic locations from March to June 2005. A multistage sampling method was used to recruit the participants from Beijing and Shanghai. Two urban districts and one rural district in both cities were chosen and the eligible candidates listed in the residential registration record were selected randomly. All participants attended a physical examination during which standard anthropometric measurements and fasting blood samples were collected. Type 2 diabetes was defined as fasting plasma glucose ≥7.0 mmol/l or previously diagnosed diabetic and receiving glucose-lowering treatment (N = 424: 37% screen-detected, 63% previously diagnosed and receiving anti-diabetic treatment) [10]. Normal fasting glucose (NFG) (N = 1,908) was defined as nondiabetic individuals having fasting glucose<5.6 mmol/l, and impaired fasting glucose (IFG) (N = 878, all were screen-detected and treatment-naive) was defined as nondiabetic individuals having 5.6≤fasting glucose<7.0 mmol/l. Homeostasis model assessment of insulin resistance (HOMA-IR) and beta-cell function (HOMA-B) was estimated by Levy's computer model [11]. Written informed consents were obtained from all participants, and study protocol was approved by the Institutional Review Board of the Institute for Nutritional Sciences.

### Genotyping

Genomic DNA was extracted from peripheral blood mononuclear cell (PBMCs) by salting-out procedure (http://protocol-online.org/prot/Detailed/3171.html). A total of nine single nucleotide polymorphisms (SNP) with a minor allele frequency (MAF) larger than 0.05 in HapMap CHB (in or near gene *MADD*, *ADRA2A*, *CRY2*, *GLIS3*, *PROX1*, *FADS1*, *C2CD4B, IGF1*, *IRS1*) were selected from the recent GWAS studies of type 2 diabetes or fasting glucose [2], [6]. Variants in or near *ADCY5*, *SLC2A2* were not genotyped because of low MAF (<5%) in HapMap CHB database. All variants were genotyped using GenomeLab SNPstream Genotyping System (Beckman Coulter) or ABI PRISM 7900HT sequence detection system (Applied Biosystems). The overall genotyping success rates were >98.8%, and the concordance rates were >98.7% in 12% of total sample (Table S1). The genotype distribution of all polymorphisms were in Hardy-Weinberg equilibrium (*P*>0.05 in whole individuals and each regional subpopulation) (Table S1).

### Statistical analyses

Hardy-Weinberg equilibrium was examined by likelihood ratio Chi-square tests. Generalized linear regression analyses were applied to test the associations of each SNP with quantitative traits. The data of HOMA-IR was natural log-transformed before analyses because of skewed distribution. Participants with known diabetes or receiving glucose-lowering treatment (N = 276) were excluded from the analyses with quantitative traits. Logistic regression was used to test the associations of each SNP with risk of diabetes and/or IFG, using individuals with NFG as reference (control) group. Glucose-increasing alleles were determined based upon the recent GWAS results [2], [6]. A recessive and dominant model was used for the SNP with glucose-increasing allele frequencies >90% (rs10885122 and rs7944585) and <10% (rs2943641), respectively, and an additive model was used for those with glucose-increasing allele frequencies ranging from 10% to 90% (rs7034200, rs11605924, rs35767, rs11071657, rs340874 and rs174550). All association analyses were performed separately in Beijing and Shanghai subpopulations and then in pooled population with adjustment firstly for sex, age, region (only for analyses in pooled population) and BMI (where appropriate) and secondly for other confounding factors including family history of diabetes, HDL, LDL, log-transformed triglyceride, lipid lowering medication, hypertension, anti-hypertensive medication. We conducted meta-analyses applying Cochran Q test in Stata to evaluate the combined effect sizes of subpopulations and the heterogeneity of effect sizes between subpopulations (Beijing vs. Shanghai).

We hypothesized that the associations between genotypes and type 2 diabetes risks were mediated by HOMA-B or HOMA-IR. The expected effect of the genotypes on the type 2 diabetes risk was therefore calculated be multiplying the magnitudes of the association between the genotypes and HOMA-B/HOMA-IR and of association between HOMA-B/HOMA-IR and type 2 diabetes risk [12]. The statistical significance between expected and observed effect was assessed using t-test. We estimated the proportion of the possible mediation of the type 2 diabetes associations by HOMA-B or HOMA-IR through dividing the expected effect by the observed effect. We also assessed the discriminatory ability of genetic variants for type 2 diabetes risk and/or IFG by calculating the area under Receiver Operating Characteristics (ROC) curve (AUC). Power calculations were performed using effect size and odd ratios reported in the original GWAS [2], [6] and sample size and glucose-increasing allele frequencies of our study with Quanto software (http://hydra.usc.edu/gxe). Statistical analyses were conducted using Stata (version 9.2; Stata, College Station, TX) or SAS (version 9.2; SAS Institute, Inc.). Two-side *P* values <0.05 were considered to be statistically significant.

## Results

Descriptive characteristics of the population are shown in Table 1. The prevalence of type 2 diabetes was high in Beijing subpopulation (17.3%) compared to Shanghai subpopulation (9.3%). Accordingly, the levels of diabetes related quantitive traits, including BMI, fasting glucose, HbA_1C_ and HOMA-B, were significantly different between two subpopulations.

**Table 1:** Characteristics of the study population.

	Beijing	Shanghai	*P* value
n (%male)	1574 (45.2)	1636 (43.5)	0.41
Age (years)	58.3±5.9	58.9±6.0	0.01
BMI (kg/m^2^)	25.2±3.7	23.7±3.3	<0.0001
Fasting glucose (mmol/l)	6.16±1.96	5.53±1.42	<0.0001
HbA_1C_ (%)	6.08 ± 1.22	5.90 ± 0.96	<0.0001
HOMA-B	100.1±44.9	120.0±46.9	<0.0001
HOMA-IR	1.82±1.56	1.74±0.86	0.67
Diabetes (%)	272 (17.3)	152 (9.3)	<0.0001
IFG (%)	579 (36.7)	293 (18.3)	<0.0001

Data are presented as mean ± SD, or n (%), unless otherwise indicated.

*P* values represent significance of the differences between individuals from Beijing and Shanghai.

Of the nine variants genotyped in this study, *GLIS3*-rs7034200 showed significant associations with fasting glucose (β = 0.07 mmol/l, *P* = 0.03) and HOMA-B (β = −3.03%, *P* = 0.009), but not with BMI, HbA1c or HOMA-IR (*P*≥0.20) (Table 2). None of the remaining eight loci (*MADD*-rs7944584, *ADRA2A*-rs10885122, *CRY2*-rs11605924, *PROX1*-rs340874, *C2CD4B*-rs11071657, *IGF1*-rs35767, *IRS1*- rs2943641 and *FADS1*-rs174550) were associated with any of glycemic traits (*P*≥0.10), while a nominal association was observed between *PROX1*-rs340874 and BMI (*P* = 0.04). Most of the associations with these quantitative traits were similar between Shanghai and Beijing subpopulations, except for the associations with BMI for *ADRA2A-*rs10885122 and *FADS1-*rs174550 (*P* for heterogeneity  = 0.005 and 0.08, respectively), and with HOMA-IR for *IRS1-*rs2943641 (*P* for heterogeneity  = 0.02) (Table S2).

**Table 2:** Associations with type 2 diabetes-related quantitative traits.

SNP	Gene	Alleles (glucose- increasing*/other)	Freq. glucose- increasing allele*	Glucose		HbA1c		HOMA-B		HOMA-IR		BMI	
				β(SE)	*P* †	β(SE)	*P* †	β(SE)	*P* †	β(SE)	*P* †	β(SE)	*P* ‡
rs7944584	*MADD*	A/T	0.97	0.11 (0.10)	0.24	0.07 (0.06)	0.25	−3.99 (3.54)	0.26	−0.008 (0.039)	0.84	−0.10 (0.28)	0.71
rs10885122	*ADRA2A*	G/T	0.92	0.04 (0.06)	0.50	0.05 (0.04)	0.26	1.38 (2.23)	0.54	0.019 (0.024)	0.45	−0.13 (0.17)	0.45
rs11605924	*CRY2*	A/C	0.77	0.03 (0.04)	0.43	−0.002 (0.03)	0.93	−1.70 (1.37)	0.22	0.003 (0.015)	0.84	−0.09 (0.11)	0.42
rs7034200	*GLIS3*	A/C	0.43	0.07 (0.03)	**0.03**	0.01 (0.02)	0.53	−3.03 (1.15)	**0.009**	−0.005 (0.013)	0.67	0.12 (0.09)	0.20
rs340874	*PROX1*	C/T	0.38	−0.04 (0.03)	0.18	−0.02 (0.02)	0.28	1.95 (1.19)	0.10	0.015 (0.013)	0.25	−0.19 (0.09)	**0.04**
rs11071657	*C2CD4B*	A/G	0.63	0.007 (0.03)	0.84	0.03 (0.02)	0.19	0.43 (1.21)	0.72	0.002 (0.013)	0.89	0.04 (0.09)	0.65
rs35767	*IGF1*	G/A	0.66	0.02 (0.03)	0.63	0.01 (0.02)	0.53	−0.13 (1.22)	0.92	0.008 (0.013)	0.57	−0.14 (0.10)	0.14
rs2943641	*IRS1*	T/C	0.07	0.01 (0.07)	0.82	−0.002 (0.04)	0.96	0.65 (2.39)	0.78	−0.006 (0.026)	0.83	−0.03 (0.19)	0.89
rs174550	*FADS1*	T/C	0.65	0.04 (0.03)	0.27	0.02 (0.02)	0.37	−0.56 (1.21)	0.65	0.007 (0.013)	0.61	0.16 (0.10)	0.09

Data are presented as β ± SE unless otherwise indicated. HOMA-IR was log-transformed before analyses.

Participants previously diagnosed with type 2 diabetes or receiving glucose-lowering treatment (n = 264) were excluded from the analyses for diabetes related traits.

A recessive and dominant model was used for the SNP with glucose-increasing allele frequencies >90% (rs10885122 and rs7944585) and <10% (rs2943641), respectively, and an additive model was used for those with glucose-increasing allele frequencies ranging from 10% to 90% (rs7034200, rs11605924, rs35767, rs11071657, rs340874 and rs174550).

*P* values <0.05 were shown in bold.

* Glucose-increasing alleles were determined based upon the recent GWAS results [2], [6].

† *P* values were adjusted for age, sex, BMI and region.

‡ *P* values were adjusted for age, sex and region.

Of the nine SNPs, *GLIS3*-rs7034200 was also significantly associated with type 2 diabetes (OR [95%CI]  = 1.27 [1.09–1.49], *P* = 0.003), IFG (OR [95%CI]  = 1.21 [1.07–1.36], P = 0.002), and combined IFG/type 2 diabetes (OR [95%CI]  = 1.23 [1.10–1.37], *P* = 0.0002) (Table 3). The glucose-increasing-(A-) allele of *CRY2*-rs11605924 was significantly associated with increased risk of combined IFG/type 2 diabetes (OR [95%CI]  =  1.15[1.01–1.30], *P* = 0.04). Although the associations with type 2 diabetes (OR [95%CI]  = 1.14 [0.94–1.37]) and IFG (OR [95%CI]  = 1.15 [1.00–1.33]) showed similar trends, the results did not reach nominal significance (*P*≥0.05). The direction of associations for loci *ADRA2A*, *IGF1* and *IRS1* were consistent to those observed in the previous GWAS [2], [6], however none of the remaining loci were associated with type 2 diabetes (*P*≥0.21), IFG (*P*≥0.10) or combined IFG/type 2 diabetes (*P*≥0.22). Power calculation suggested that our study had less than 25% power to detect the previously reported OR and beta values in white Europeans [2], [6] at *P*<0.05. Notably, further adjustment for family history of diabetes, lipid profile, medication information and hypertension did not materially change the associations of *GLIS3*-rs7034200 with HOMA-B and type 2 diabetes, but abolished the association between *CRY2* and combined IFG/type 2 diabetes (Table S3).

**Table 3:** Associations with type 2 diabetes and combined IFG/type 2 diabetes

SNP	Gene	Alleles (glucose increasing*/other)	T2DM/IFG/NFG	Freq. glucose- increasing allele* (T2DM/IFG/NFG)	T2DM vs. NFG		IFG vs. NFG		Combined IFG/T2DMvs. NFG	
					OR(95%CI)	*P*	OR(95%CI)	*P*	OR(95%CI)	*P*
rs7944584	*MADD*	A/T	424/8771902	0.96/0.98/0.97	1.00 (0.63–1.58)	0.99	1.39 (0.94–2.05)	0.10	1.23 (0.88–1.71)	0.22
rs10885122	*ADRA2A*	G/T	423/878/1893	0.93/0.91/0.92	1.23 (0.89–1.79)	0.21	0.86 (0.69–1.08)	0.20	0.96 (0.78–1.18)	0.70
rs11605924	*CRY2*	A/C	423/872/1884	0.78/0.78/0.77	1.14 (0.94–1.37)	0.18	1.15 (1.00–1.33)	0.05	1.15 (1.01–1.30)	**0.04**
rs7034200	*GLIS3*	A/C	422/870/1881	0.47/0.46/0.41	1.27 (1.09–1.49)	**0.003** †	1.21 (1.07–1.36)	**0.002** †	1.23 (1.10–1.37)	**0.0002** †
rs340874	*PROX1*	C/T	423/869/1887	0.35/0.38/0.38	0.91 (0.77–1.07)	0.26	1.02 (0.90–1.15)	0.76	0.98 (0.88–1.09)	0.68
rs11071657	*C2CD4B*	A/G	422/877/1889	0.62/0.62/0.63	0.94 (0.80–1.10)	0.43	0.96 (0.85–1.08)	0.48	0.95 (0.85–1.06)	0.39
rs35767	*IGF1*	G/A	424/875/1899	0.67/0.66/0.66	1.07 (0.91–1.26)	0.44	1.03 (0.91–1.16)	0.67	1.04 (0.93–1.16)	0.49
rs2943641	*IRS1*	T/C	422/873/1895	0.08/0.06/0.07	1.16 (0.85–1.57)	0.35	0.92 (0.71–1.18)	0.49	1.01 (0.81–1.26)	0.93
rs174550	*FADS1*	T/C	419/873/1886	0.67/0.68/0.63	0.99 (0.84–1.16)	0.90	1.04 (0.92–1.18)	0.53	1.03 (0.92–1.15)	0.66

Data are presented as OR (95%CI) unless otherwise indicated. *P* values were adjusted for age, sex, BMI and region.

A recessive and dominant model was used for the SNP with glucose-increasing allele frequencies >90% (rs10885122 and rs7944585) and <10% (rs2943641), respectively, and an additive model was used for those with glucose-increasing allele frequencies ranging from 10% to 90% (rs7034200, rs11605924, rs35767, rs11071657, rs340874 and rs174550).

*P* values <0.05 were shown in bold.

*Glucose-increasing alleles were determined based upon the recent GWAS results [2], [6]

† The associations remained significant after Bonferroni correction for multiple tests, and the Bonferroni corrected cutoff *P* value is 0.006 (0.05/9 tests).

The ROC_AUC_ values of *GLIS*3-rs7034200 and *CRY2-*rs11605924 for the discrimination of type 2 diabetes were 0.543 and 0.511 respectively. While the combination of all nine SNPs significantly (*P* = 0.01) improved ROC_AUC_ (ROC_AUC_ = 0.574)_,_ the discriminative accuracy of type 2 diabetes risk remains low (Table S4).

To examine whether impaired beta-cell function (HOMA-B) or insulin resistance (HOMA-IR) mediates the associations of *GLIS*3-rs7034200 with type 2 diabetes and/or combined IFG/type 2 diabetes, we re-evaluated the associations in multivariate model (adjusting for family history of diabetes, lipid profile, medication information, hypertension and life-style factors) with further adjustment for HOAM-B or HOMA-IR. However, the associations with type 2 diabetes and/or combined IFG/type 2 diabetes were abolished or attenuated; i.e. OR for type 2 diabetes: 1.19 [0.96–1.49]), *P* = 0.11; and OR for IFG/type 2 diabetes: 1.15 [1.01–1.31], *P* = 0.04) after adjusting for HOMA-B, whereas the associations remained unchanged after further adjustment for HOMA-IR (Table S5). We then performed Mendelian randomization analysis to test whether and to what extent the diabetes association might be attributable to impaired beta-cell function (HOMA-B). We found that the expected effect (β = 0.15), estimated by multiplying the magnitude of the association between *GLIS3*-rs7034200 and HOMA-B and of the association between HOMA-B and type 2 diabetes risk, was comparable to the observed effect (β = 0.24, *P*_(exp/obs)_ = 0.53). HOMA-B explained 63% of the association between *GLIS3*-rs7034200 and type 2 diabetes, calculated by comparing the expected effect with the observed effect (Table 4).

**Table 4:** Results of Mendelian randomization analyses for the associations of *GLIS3*-rs7034200 with type 2 diabetes, IFG and combined IFG/type 2 diabetes.

	β1*	β2†	β obs‡	β exp§	*P* (obs/exp)||	Proportion(%)¶
Via HOMA-B						
Type 2 diabetes	−0.06 (0.02)	−2.42 (0.12)	0.24 (0.08)	0.15 (0.12)	0.53	63
IFG	−0.06 (0.02)	−1.03 (0.07)	0.19 (0.06)	0.06 (0.07)	0.18	34
Combined IFG/type 2 diabetes	−0.06 (0.02)	−1.30 (0.06)	0.21(0.05)	0.08 (0.06)	0.10	38
Via HOMA-IR						
Type 2 diabetes	0.016 (0.023)	0.78 (0.08)	0.24 (0.08)	0.01 (0.08)	0.04	4.2
IFG	0.016 (0.023)	0.55 (0.07)	0.19 (0.06)	0.009 (0.04)	0.01	4.8
Combined IFG/type 2 diabetes	0.016 (0.023)	0.64 (0.06)	0.21(0.05)	0.01 (0.06)	0.01	4.8

Data on HOMA-B and log-transformed HOMA-IR were standardized to z-score before analyses and effect size are presented as standardized βs (z-score).

* β1: the effect size of association between *GLIS3*-rs7034200 and HOMA-B (or HOMA-IR).

† β2: the effect size of association between HOMA-B (or HOMA-IR) and type 2 diabetes (or IFG or combined IFG/type 2 diabetes).

‡ β(obs): Observed effect size of associations between *GLIS3*-rs7034200 and type 2 diabetes (or IFG or combined IFG/type 2 diabetes).

§ β(exp): Expected effect of associations between variants and type 2 diabetes (or IFG or combined IFG/type 2 diabetes) via HOMA-B (or HOMA-IR), calculated by β1×β2.

|| *P* (obs/exp): *P*-value for difference between β (obs) and β (exp).

¶ Proportion: The proportion of associations between *GLIS3*-rs7034200 and type 2 diabetes (or IFG or combined IFG/type 2 diabetes) that could be explained by the association with HOMA-B (or HOMA-IR), calculated by β (exp)/β (obs).

## Discussion

In this population-based cohort of Chinese Hans, we confirmed the associations of *GLIS3*-rs7034200 with fasting glucose and beta-cell function as estimated by HOMA-B, as well as with risk of type 2 diabetes and/or IFG. Further analyses suggested that association between *GLIS3*-rs7034200 and type 2 diabetes was mediated by HOMA-B. We also observed a moderate association between *CRY2*-rs11605924 and combined IFG/type 2 diabetes. Although the associations between the SNPs in *MADD*, *ADRA2A*, *C2CD4B*, *IGF1, IRS1*and *FADS1* with fasting glucose or type 2 diabetes were directionally consistent with those observed for white Europeans and of similar magnitude to that observed in the previous GWAS, none reached nominal significance [6].

In whites of European ancestry, *GLIS3* was identified as in GWA studies for fasting glucose and beta-cell function as estimated by HOMA-B, yet the locus was also nominally associated with type 2 diabetes [6]. In the present study of Chinese Hans, *GLIS3*-rs7034200 was significantly associated not only with fasting glucose and HOMA-B, but also with type 2 diabetes risk. Consistent with our results, a recent study in case-control samples of Chinese Hans also observed a significant association between *GLIS3*-rs7034200 and type 2 diabetes [8]. Interestingly, the observed OR for type 2 diabetes risk in this study was significantly larger than those reported previously for white Europeans [6] (1.27 vs. 1.03, *P* = 0.009). Since *GLIS3*-rs7034200 is more likely a marker of the causal variant and the risk A-allele frequency of *GLIS3*-rs7034200 is similar between Chinese Hans and white Europeans, the disparity in the magnitude of the associations with glucose and type 2 diabetes might be due to ethnic-specific LD pattern between *GLIS3*-rs7034200 and the as-yet unidentified causal variant. Nevertheless, we cannot rule out the possibility that the observed effect size might be population-specific or overestimated due to the “winner's curse”. Recently, a large study in middle-aged Danish population reported that *GLIS3*-rs7034200 was associated with glucose-stimulated insulin response, and 57% of its effect on fasting glucose could be ascribed to association with insulin release (BIGTT-AIR) [7]. Consistent with their observation, we found that impaired beta-cell function estimated by HOMA-B explained about 63% of the association between *GLIS3*-rs7034200 and type 2 diabetes, and further adjustment for HOMA-B abolished this association. These findings are also consistent with the evidence from *in vivo* and in vitro studies that suggest for a critical role of Glis3 in the regulation of pancreatic beta-cell development and insulin gene expression [13]. Since HOMA-B explained only 63% of the association between *GLIS3*-rs7034200 and type 2 diabetes, additional unknown mechanisms independent of insulin release might also mediate the association.

For *CRY2*-rs11605924 variant, we observed a moderate association with combined IFG/type 2 diabetes, but not with type 2 diabetes risk or its related traits. Since the association of *CRY2*-rs11605924 with combined IFG/type 2 diabetes was abolished after further adjustment for other confounding factors including family history of diabetes, lipid profile, medication information and hypertension, this result should be interpreted with caution. We also failed to replicate the associations of other seven SNPs with fasting glucose and other diabetes-related traits. For these loci (*ADRA2A*, *CRY2*, *MADD*, *IGF1*and *FADS1*), the associations with fasting glucose were directionally consistent with those previously reported by the original study with comparable effect sizes [6]. A possible reason for our failure to completely confirm formerly identified associations of these variants is that genetic variants might exhibit their effects in the setting of a specific environment; hence the inconsistence might be induced by the population difference in environment factors. Certainly, given that we have less than 25% power to detect the previously reported beta values for fasting glucose or ORs for type 2 diabetes at *P*<0.05 (Table S1), we can not rule out the possibility that it might be due to insufficient power of our study. Differences in allele frequencies and LD patterns between Chinese Hans and Europeans are also possible reasons. The beta values for the associations between *PROX1-*rs340874 and fasting glucose and between *IRS1-*rs2943641 and HOMA-IR were in the opposite direction to those reposted in previous GWAS [2], [6], but these associations were not significant. Studies with larger sample size are needed to draw a more definite conclusion. Notably, we observed a significant difference in genotype distribution of *FADS1*-rs174550 between Shanghai and Beijing subpopulations. Consistent with our results, a previous study also observed that two SNPs near *FADS1*-rs174550 (∼25 kb) showed significant difference in allele frequencies between the northern and southern Hans cluster [14]. Therefore, it is possible that the genetic architecture of *FADS1* gene might be different between the Northern and Southern Chinese Hans. Additionally, the associations of *MADD*, *ADRA2A*, *IRS1* and *FASD1* with type 2 diabetes or its related traits were directional different between two subpopulations. The discrepancies might be partially explained by the geographic differences in diabetes prevalence which may due to sampling, although further studies are clearly needed to clarify this.

In conclusion, our results confirmed the associations of *GLIS3*-rs7034200 with fasting glucose and risk of type 2 diabetes, and we also observed a moderate significant association between *CRY2*-rs11605924 and combined IFG/type 2 diabetes in Chinese Hans. The *GLIS3*-rs7034200 exhibited larger effect size on type 2 diabetes compared to that in white Europeans, and its contribution to type 2 diabetes risk is mainly mediated through impaired beta-cell function.

## Supporting Information

Table S1 Genotype distribution by sex and subpopulation and statistical power.
(DOC)

Table S2 Associations with type 2 diabetes-related quantitative traits, type 2 diabetes, IFG and combined IFG/type 2 diabetes in Beijing and Shanghai subpopulations.
(XLS)

Table S3 Associations with type 2 diabetes-related quantitative traits, type 2 diabetes, IFG and combined IFG/type 2 diabetes in multivariate model.
(XLS)

Table S4 Area under for Receiver Operating Characteristics (ROC) curve (AUC) calculation with multiple SNPs and conventional type 2 diabetes risk factors.
(DOC)

Table S5 Associations of *GLIS3*-rs7034200 with type 2 diabetes, IFG and combined IFG/type 2 diabetes in multiple adjustment models.
(DOC)
