# Supplementary material for: Variants in GLIS3 and CRY2 Are Associated with Type 2 Diabetes and Impaired Fasting Glucose in Chinese Hans
Source: PLoS One. 2011 Jun 29;6(6):e21464. doi: 10.1371/journal.pone.0021464 (PMC3126830; doi:10.1371/journal.pone.0021464)
Supplement: Table S1 — Genotype distribution by sex and subpopulation and statistical power. (DOC) [file pone.0021464.s001.doc]

Supplementary Table 1. Genotype distribution by sex and subpopulation and statistical power

| SNP | Gene | Alleles  (glucose-  increasing/other) | Freq. glucose-  increasing allele  (BJ/SH/ HapMap-CHB/ GWAS[1,2]) | HWE | | Call  rate  (%) | Concor-dance rate  (%) | *P* for genotype distribution between sex | *P* for genotype distribution between subpopulations | Power † | |
| --- | --- | --- | --- | --- | --- | --- | --- | --- | --- | --- | --- |
|  |  |  |  | BJ | SH |  |  |  |  | Glucose | T2DM |
| rs7944584 | *MADD* | A/T | 0.97/0.97 /0.95 /0.75 | 0.81 | 0.70 | 99.8 | 99.0 | 0.13 | 0.70 | 0.05 | NA |
| rs10885122 | *ADRA2A* | G/T | 0.91/0.92/ 0.92/0.87 | 0.65 | 0.27 | 99.1 | 99.0 | 0.28 | 0.36 | 0.06 | 0.06 |
| rs11605924 | *CRY2* | A/C | 0.76/0.79/ 0.76/ 0.49 | 0.78 | 0.71 | 99.0 | 99.5 | 0.19 | 0.09 | 0.06 | 0.07 |
| rs7034200 | *GLIS3* | A/C | 0.43/0.44/ 0.30/ 0.49 | 0.87 | 0.31 | 98.8 | 99.0 | 0.80 | 0.45 | 0.07 | 0.07 |
| rs340874 | *PROX1* | C/T | 0.38/0.37/0.33/0.52 | 0.26 | 0.41 | 99.0 | 99.2 | 0.54 | 0.27 | 0.06 | 0.14 |
| rs11071657 | *C2CD4B* | A/G | 0.62/0.63/0.68/0.63 | 0.61 | 0.73 | 99.6 | 99.7 | 0.28 | 0.60 | 0.05 | 0.07 |
| rs35767 | *IGF1* | G/A | 0.66/ 0.65/0.66/ 0.85 | 0.41 | 0.81 | 99.6 | 99.2 | 0.24 | 0.59 | 0.06 | 0.08 |
| rs2943641 | *IRS1* | T/C | 0.07/0.06/0.07/0.39 | 0.76 | 0.76 | 99.4 | 99.5 | 0.20 | 0.27 | NA | 0.23 |
| rs174550 | *FADS1* | T/C | 0.71 /0.58/0.66/ 0.64 | 0.33 | 0.78 | 99.0 | 98.7 | 0.84 | <0.0001 | 0.06 | 0.08 |

HWE, Hardy-Weinberg equilibrium; BJ, Beijing; SH, Shanghai.

* Glucose-increasing alleles were determined based upon the recent GWAS results [1,2]

† The statistical power shown in the table were calculated for association of each SNP with fasting glucose and type 2 diabetes using the effect size or odds ratio reported in the original studies [1,2] and sample size and coded allele frequencies in our own study under an additive model.

**Reference**

1. Dupuis J, Langenberg C, Prokopenko I, Saxena R, Soranzo N, et al. (2010) New genetic loci implicated in fasting glucose homeostasis and their impact on type 2 diabetes risk. Nat Genet 42: 105-116.

2. Rung J, Cauchi S, Albrechtsen A, Shen L, Rocheleau G, et al. (2009) Genetic variant near IRS1 is associated with type 2 diabetes, insulin resistance and hyperinsulinemia. Nat Genet 41: 1110-1115.
