# Supplementary material for: Variants in GLIS3 and CRY2 Are Associated with Type 2 Diabetes and Impaired Fasting Glucose in Chinese Hans
Source: PLoS One. 2011 Jun 29;6(6):e21464. doi: 10.1371/journal.pone.0021464 (PMC3126830; doi:10.1371/journal.pone.0021464)
Supplement: Table S4 — Area under for Receiver Operating Characteristics (ROC) curve (AUC) calculation with multiple SNPs and conventional type 2 diabetes risk factors. (DOC) [file pone.0021464.s004.doc]

**Supplementary Table 4**. Area under for Receiver Operating Characteristics (ROC) curve (AUC) calculation with multiple SNPs and conventional type 2 diabetes risk factors

|  | AUC (95%CI) | | |
| --- | --- | --- | --- |
|  | Type 2 diabetes | IFG | Combined IFG./type 2 diabetes |
| *GLIS3*-rs7034200 | 0.543 (0.515-0.571) | 0.531 (0.509-0.552) | 0.536 (0.517-0.555) |
| *CRY2-* rs11605924 | 0.511 (0.485-0.537) | 0.508 (0.487-0.528) | 0.510 (0.492-0.527) |
| All 9 SNPs Combined | 0.574 (0.543-0.604)‡ | 0.563 (0.540-0.587)‡ | 0.564 (0.543-0.585)‡ |
| Conventional risk factors * | 0.789 (0.766-0.812) | 0.714 (0.693-0.734) | 0.729 (0.710-0.746) |
| Conventional risk factors plus 9 SNPs | 0.792 (0.768-0.815) | 0.719 (0.698-0.739) | 0.732 (0.715-0.750) † |

*Conventional risk factors included age, sex, region, BMI, family history of diabetes, smoking, alcohol use, physical activity, HDL, LDL, log-transformed triacylglycerol, hypertension, lipid lowering medication and anti-hypertensive medication.

† *P*<0.05 compared with model based on conventional risk factors.

‡ *P*<0.05 compared with model base on *GLIS3*-rs7034200
