# Supplementary material for: Variants in GLIS3 and CRY2 Are Associated with Type 2 Diabetes and Impaired Fasting Glucose in Chinese Hans
Source: PLoS One. 2011 Jun 29;6(6):e21464. doi: 10.1371/journal.pone.0021464 (PMC3126830; doi:10.1371/journal.pone.0021464)
Supplement: Table S5 — Associations of GLIS3-rs7034200 with type 2 diabetes, IFG and combined IFG/type 2 diabetes in multiple adjustment models. (DOC) [file pone.0021464.s005.doc]

**Supplementary Table 5. Associations of *GLIS3*-rs7034200 with type 2 diabetes, IFG and combined IFG/type 2 diabetes** in multiple adjustment models.

|  | Model 1 | | Model 2 | | Model 3 | |
| --- | --- | --- | --- | --- | --- | --- |
|  | OR (95%CI) | *P* | OR (95%CI) | *P* | OR (95%CI) | *P* |
| *GLIS3*-rs7034200 |  |  |  |  |  |  |
| Type 2 diabetes | 1.25 (1.05-1.47) | 0.01 | 1.19 (0.96-1.49) | 0.11 | 1.26 (1.06-1.50) | 0.008 |
| IFG | 1.20 (1.06-1.35) | 0.004 | 1.15 (1.01-1.31) | 0.04 | 1.21 (1.07-1.37) | 0.003 |
| Combined IFG/type 2 diabetes | 1.21 (1.09-1.35) | 0.0006 | 1.15 (1.01-1.30) | 0.03 | 1.22 (1.09-1.37) | 0.0004 |

Data are OR (95%CI) using an additive models.

Model 1: adjusted age, sex, BMI, region, family history of diabetes, HDL, LDL, log-transformed Triglyceride, lipid lowering medication, hypertension, anti hypertensive medication, smoking, drinking and physical activity.

Model 2: Adjusted variables in model 1 plus HOMA-B

Model 3: Adjusted variables in model 1 plus HOMA-IR
